# Supplementary material for: USP28 deletion and small-molecule inhibition destabilizes c-MYC and elicits regression of squamous cell lung carcinoma
Source: eLife. 2021 Oct 12;10:e71596. doi: 10.7554/eLife.71596 (PMC8553340; doi:10.7554/eLife.71596)
Supplement: Figure 4—source data 2. [file elife-71596-fig4-data2.pptx]

## Slide 1
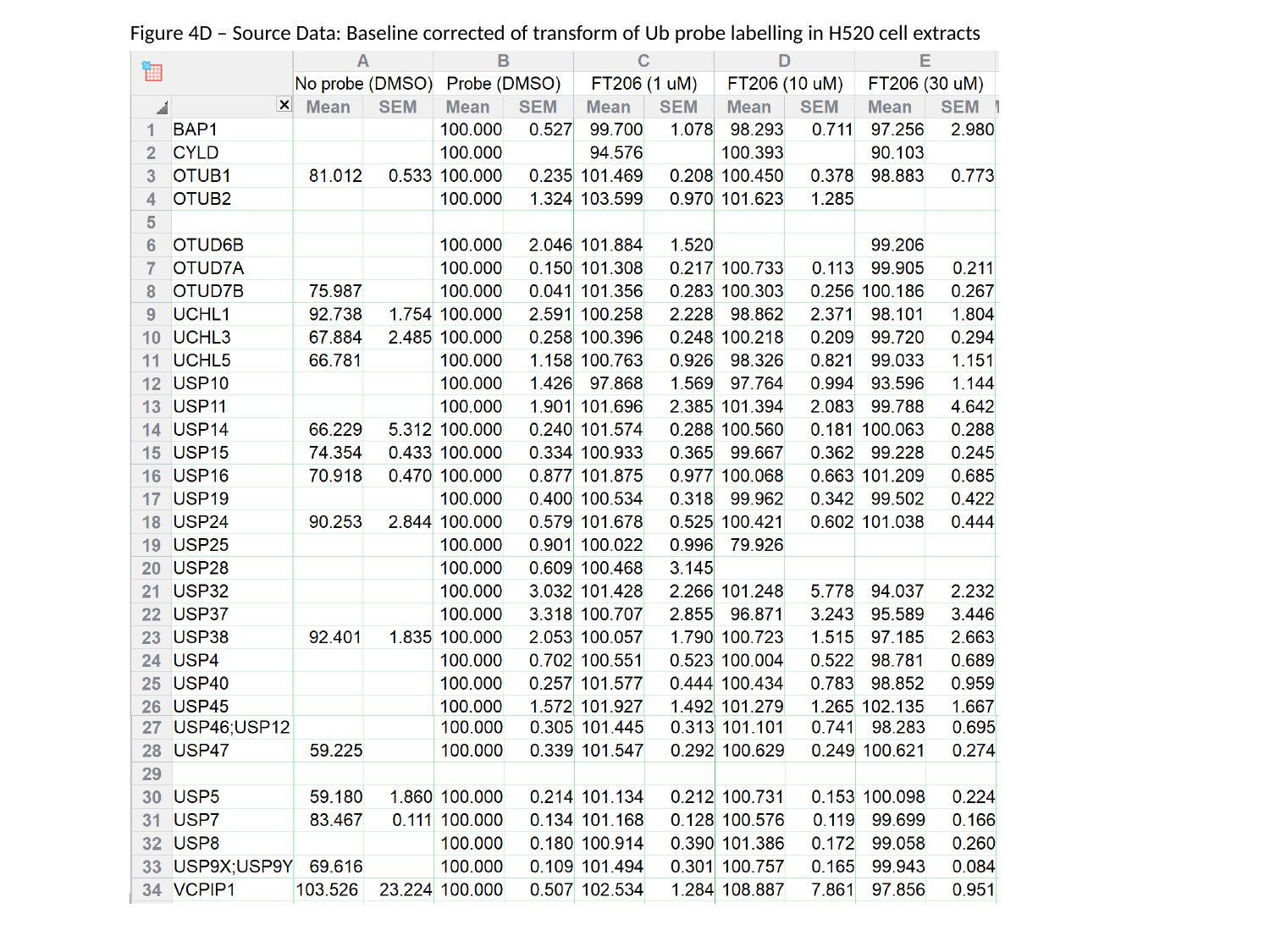

Figure 4D – Source Data: Baseline corrected of transform of Ub probe labelling in H520 cell extracts
