# Supplementary material for: USP28 deletion and small-molecule inhibition destabilizes c-MYC and elicits regression of squamous cell lung carcinoma
Source: eLife. 2021 Oct 12;10:e71596. doi: 10.7554/eLife.71596 (PMC8553340; doi:10.7554/eLife.71596)
Supplement: Source data 1. [file elife-71596-supp1.zip › All_Fig_Boxes5.pdf]

H

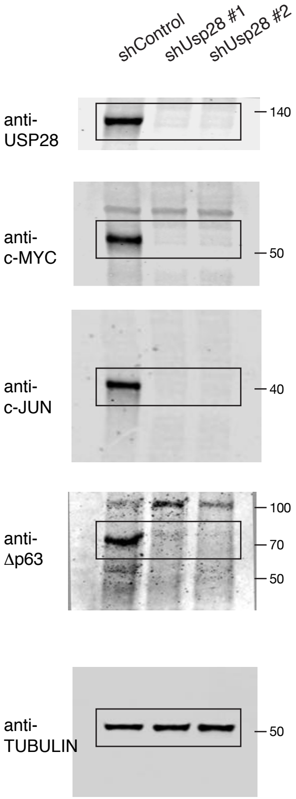

shControl
shUsp28 #1
shUsp28 #2
anti-
USP28
140
anti-
c-MYC
50
anti-
c-JUN
40
anti-
Δp63
100
70
50
anti-
TUBULIN
50

Figure 1

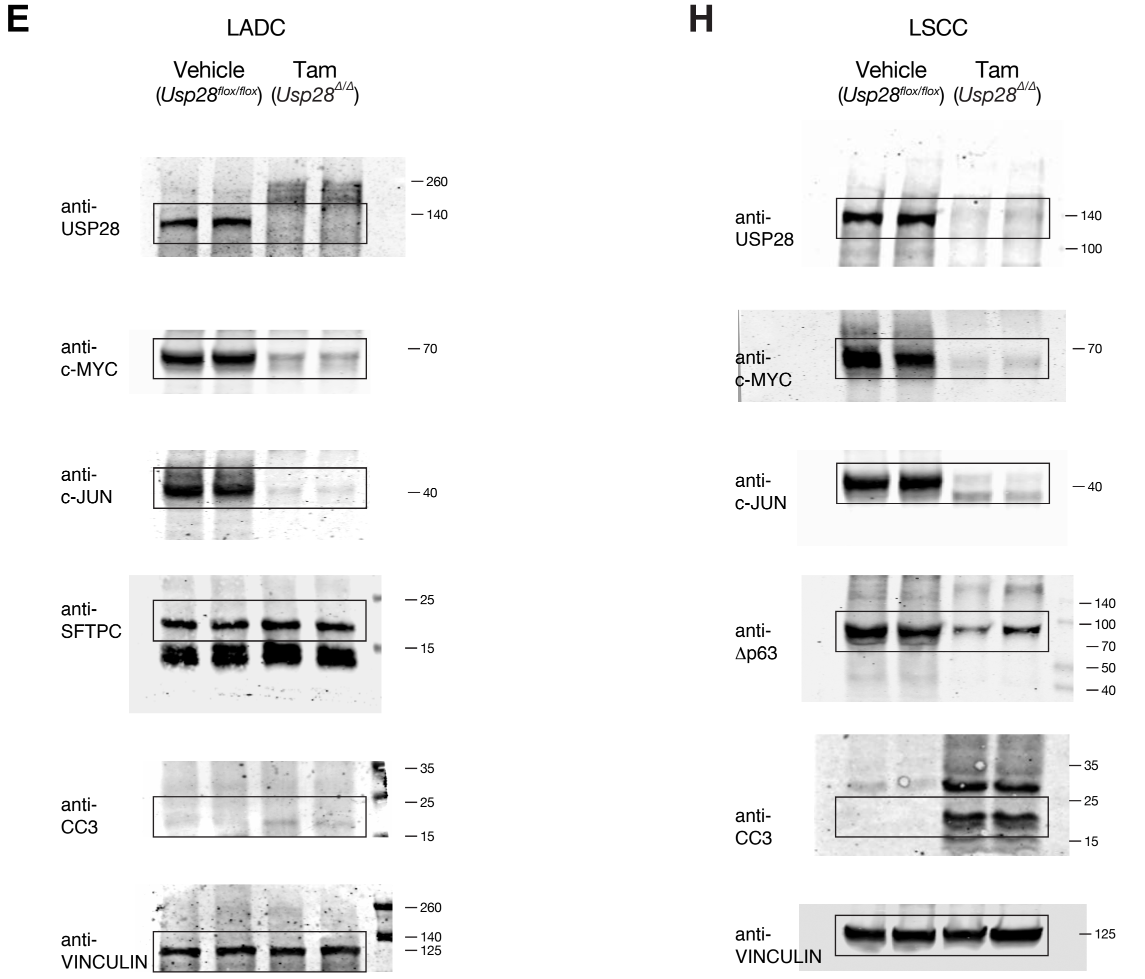

E
LADC
Vehicle (Usp28flox/flox)
Tam (Usp28Δ/Δ)
anti-USP28
anti-c-MYC
anti-c-JUN
anti-SFTPC
anti-CC3
anti-VINCULIN
260
140
70
40
25
15
35
125
H
LSCC
anti-Δp63
100
50

Figure 2

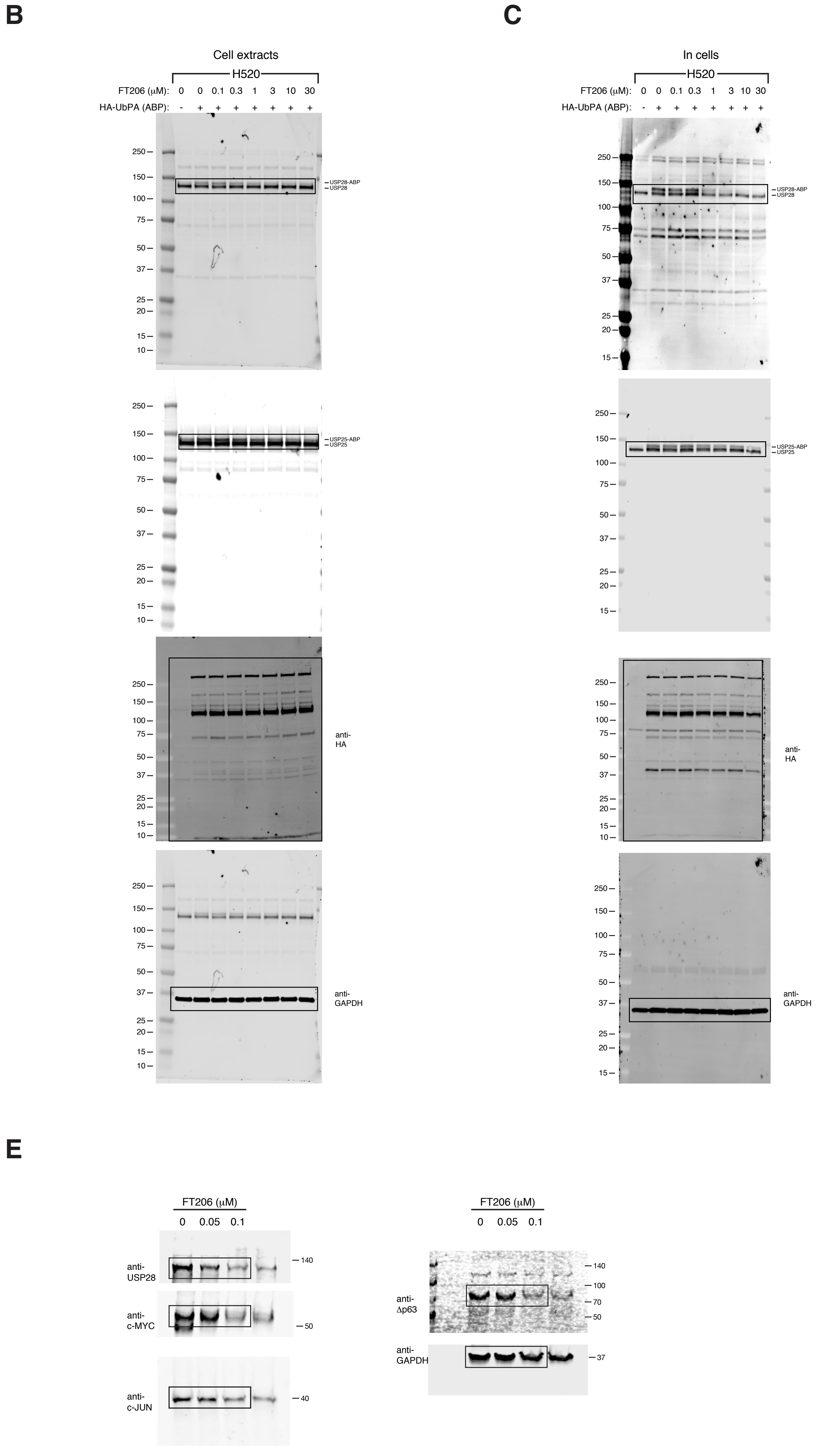
B
Cell extracts
H520
FT206 (μM): 0 0 0.1 0.3 1 3 10 30
HA-UbPA (ABP): - + + + + + + +
250
150
100
75
50
37
25
20
15
10
USP28-ABP
USP28
USP25-ABP
USP25
anti-HA
anti-GAPDH
C
In cells
H520
FT206 (μM): 0 0 0.1 0.3 1 3 10 30
HA-UbPA (ABP): - + + + + + + +
USP28-ABP
USP28
USP25-ABP
USP25
anti-HA
anti-GAPDH
E
FT206 (μM)
0 0.05 0.1
anti-USP28
anti-c-MYC
anti-c-JUN
140
50
40
anti-Δp63
anti-GAPDH
140
100
70
50
37

Figure 4

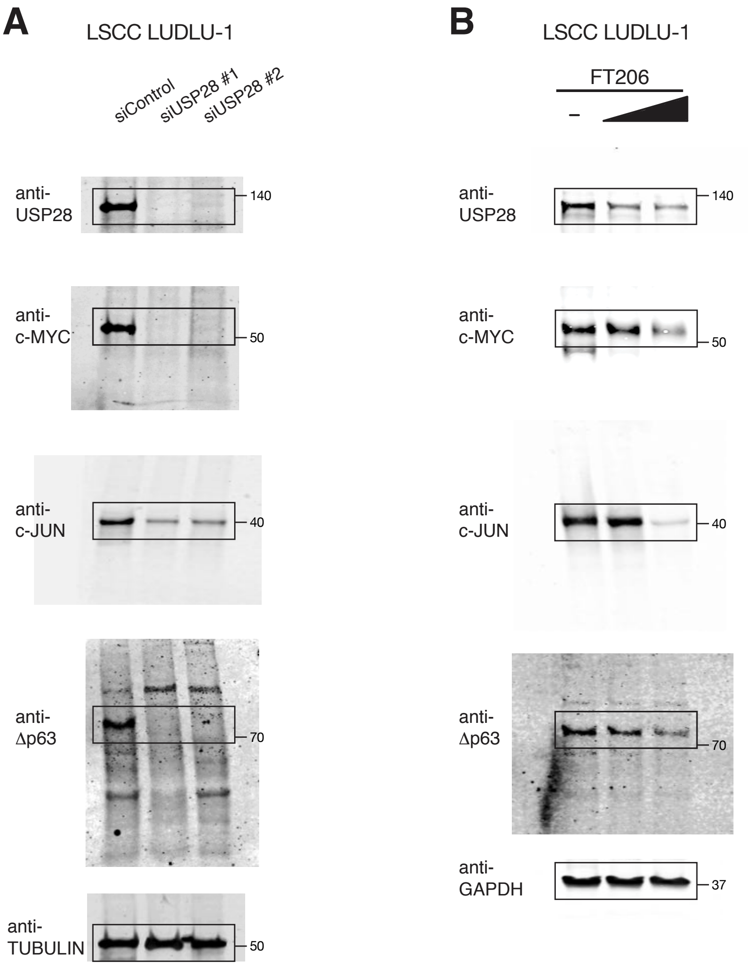
A
LSCC LUDLU-1
siControl
siUSP28 #1
siUSP28 #2
anti-
USP28
140
anti-
c-MYC
50
anti-
c-JUN
40
anti-
Δp63
70
anti-
TUBULIN
50
B
LSCC LUDLU-1
FT206
–
anti-
USP28
140
anti-
c-MYC
50
anti-
c-JUN
40
anti-
Δp63
70
anti-
GAPDH
37

Figure 6

A

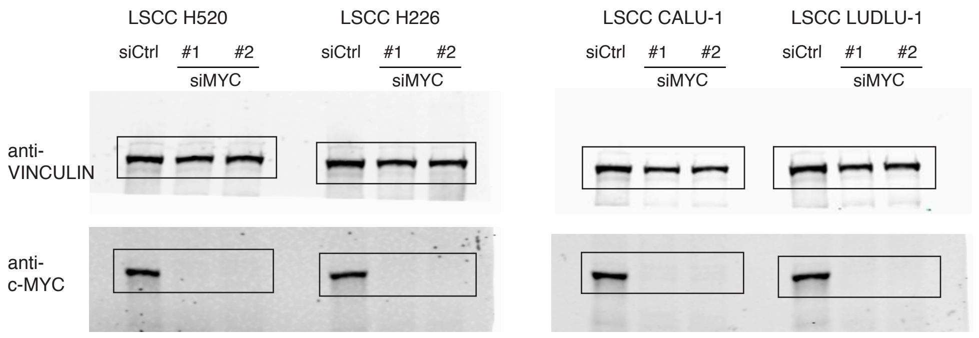

B

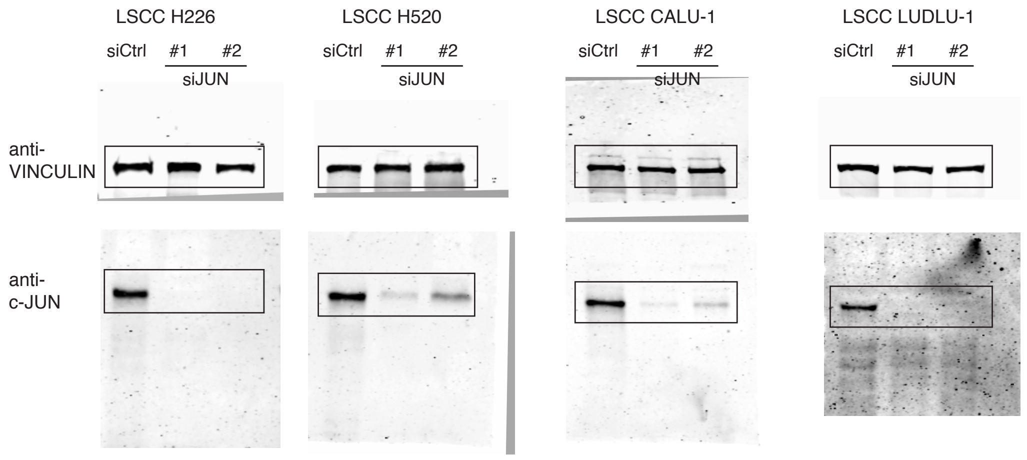

C

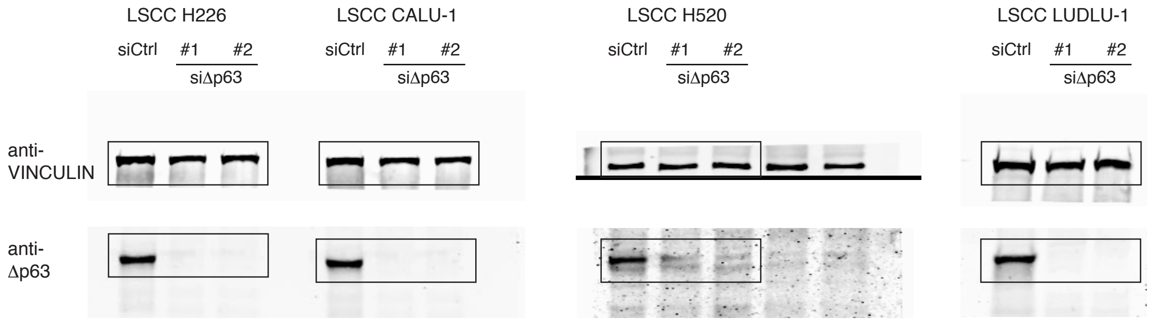

Figure 1 – figure supplement 1

A

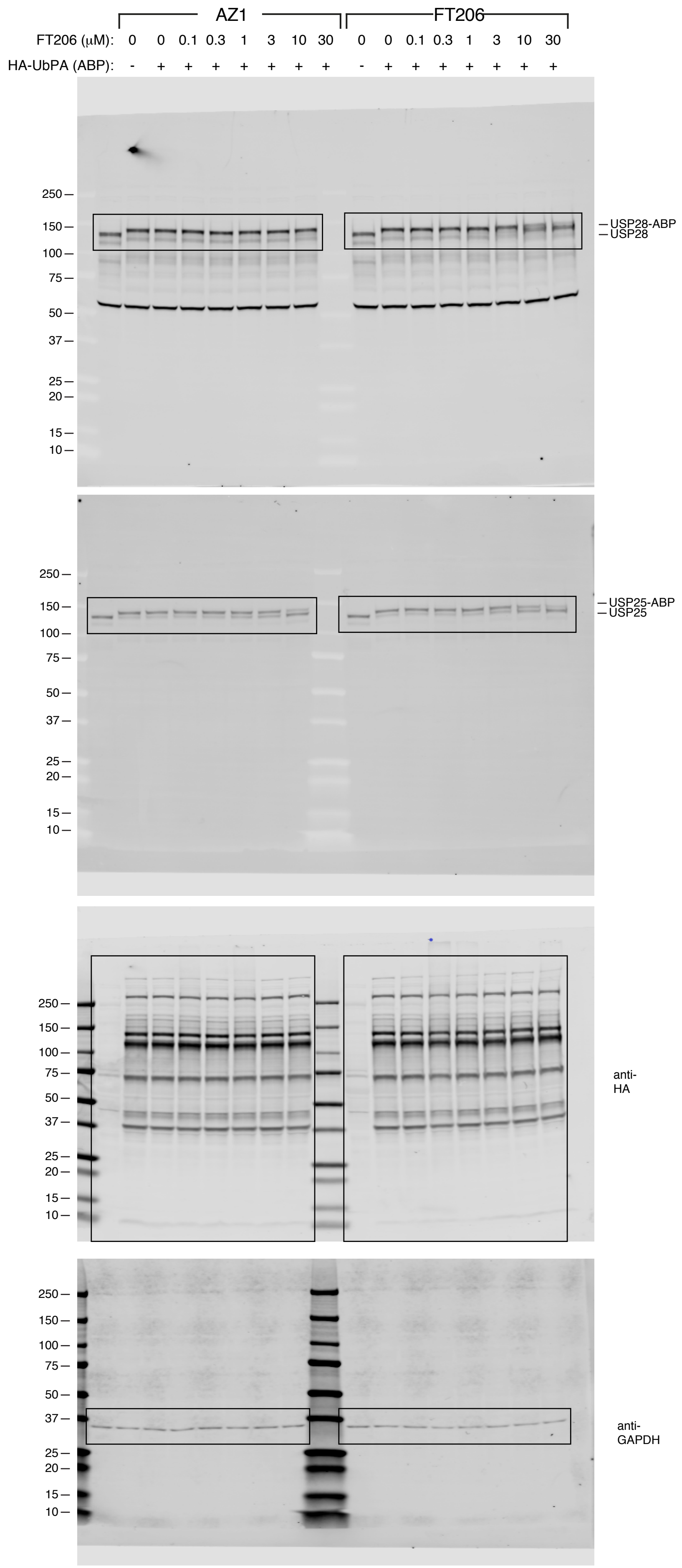

Figure 4 – figure supplement 1

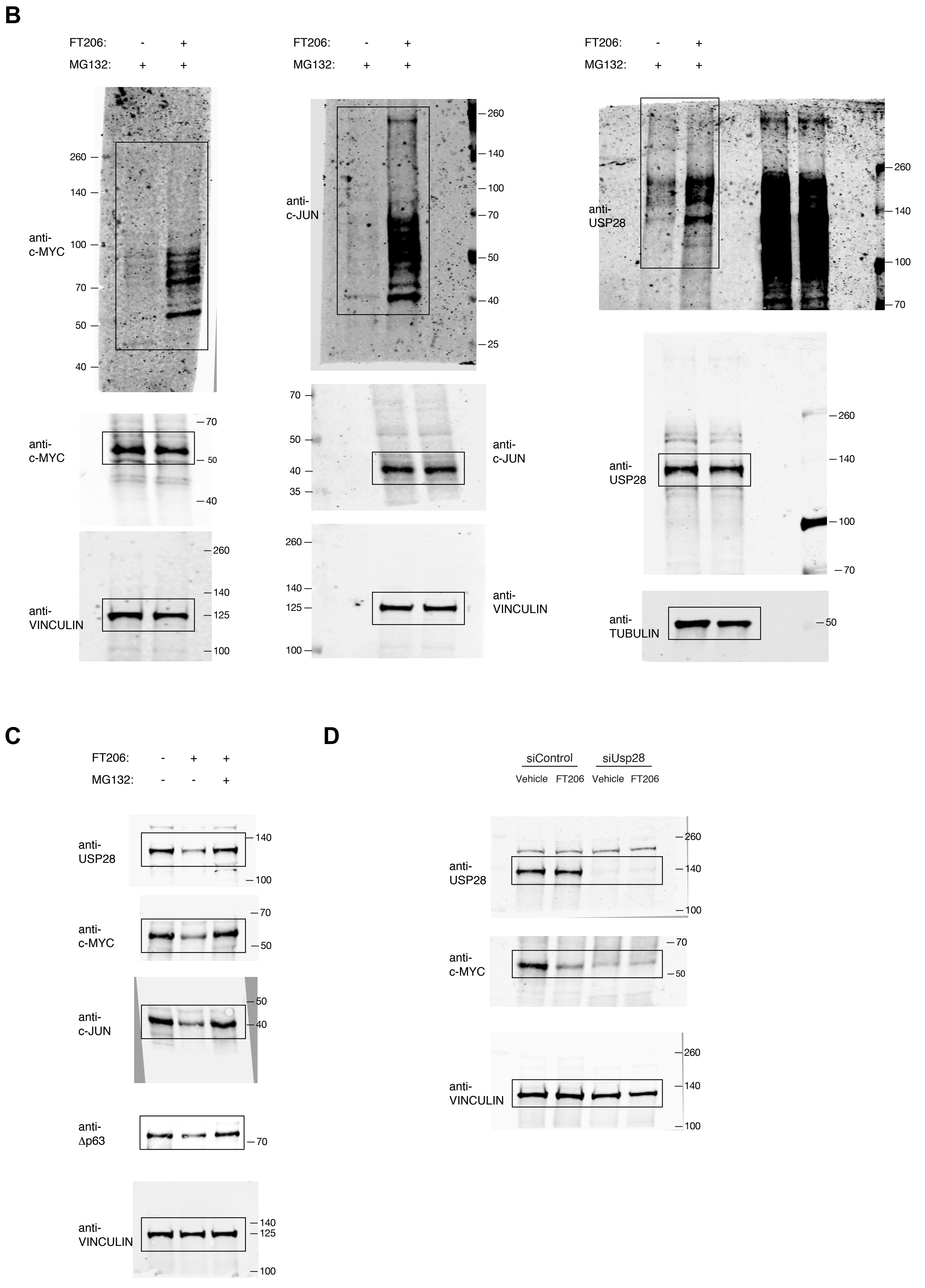

Figure 4 – figure supplement 1

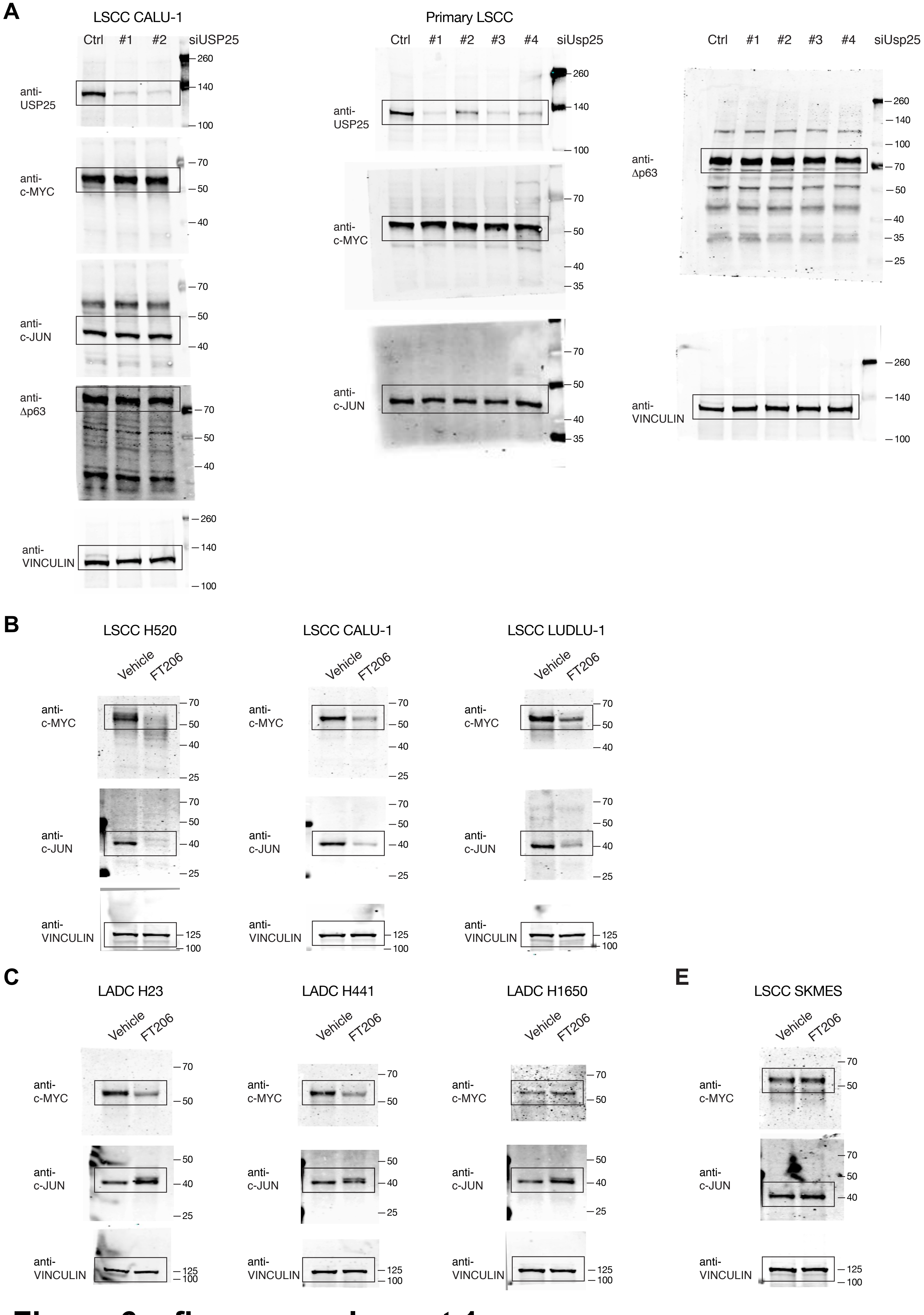

Figure 6 – figure supplement 1
