# Supplementary material for: USP28 deletion and small-molecule inhibition destabilizes c-MYC and elicits regression of squamous cell lung carcinoma
Source: eLife. 2021 Oct 12;10:e71596. doi: 10.7554/eLife.71596 (PMC8553340; doi:10.7554/eLife.71596)
Supplement: Source data 1. [file elife-71596-supp1.zip › All_Fig_NoBoxes5.pdf]

H

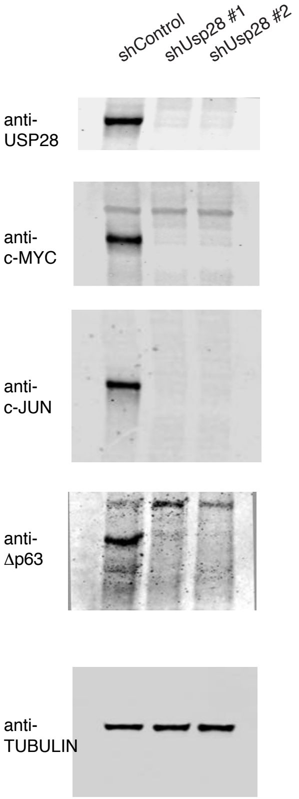

shControl
shUsp28 #1
shUsp28 #2
anti-
USP28
anti-
c-MYC
anti-
c-JUN
anti-
Δp63
anti-
TUBULIN

Figure 1

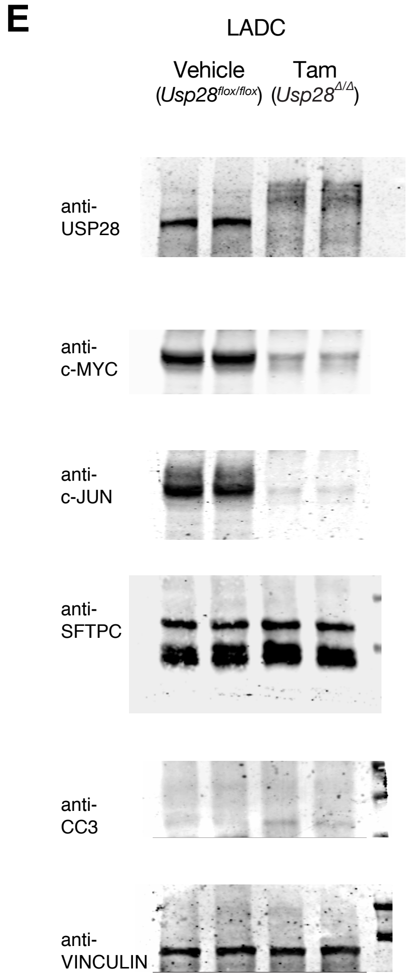
E
LADC
Vehicle
(Usp28flox/flox)
Tam
(Usp28Δ/Δ)
anti-
USP28
anti-
c-MYC
anti-
c-JUN
anti-
SFTPC
anti-
CC3
anti-
VINCULIN

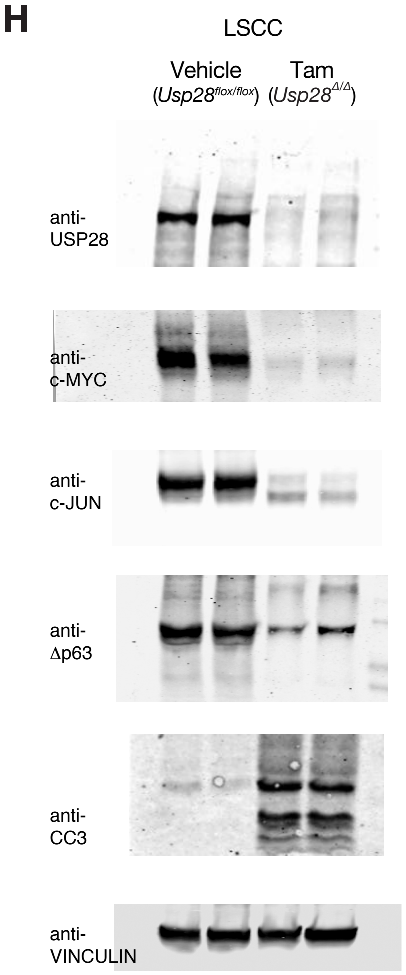
H
LSCC
Vehicle
(Usp28flox/flox)
Tam
(Usp28Δ/Δ)
anti-
USP28
anti-
c-MYC
anti-
c-JUN
anti-
Δp63
anti-
CC3
anti-
VINCULIN

Figure 2

B

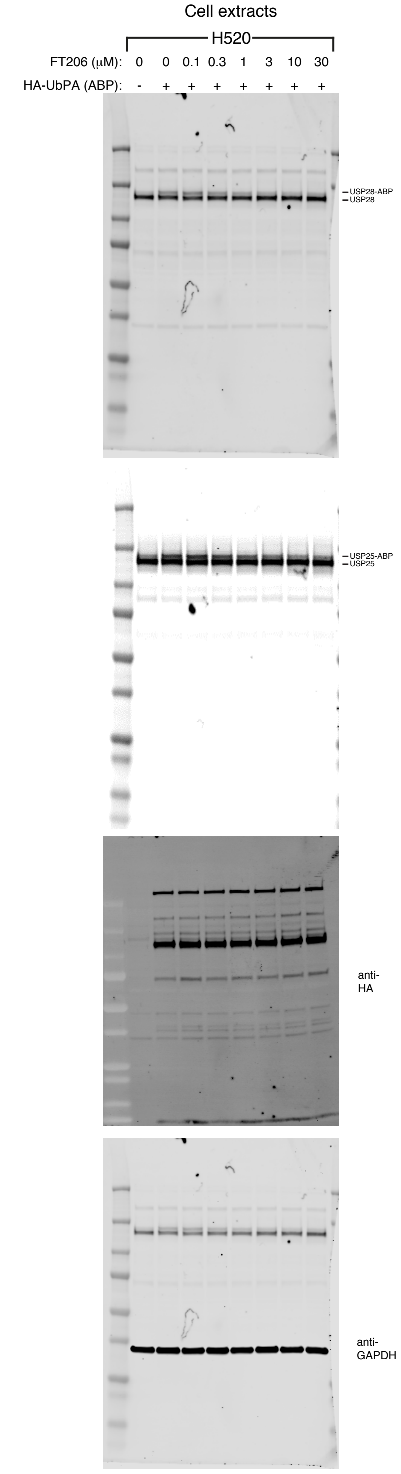
Cell extracts
H520
FT206 (μM): 0 0 0.1 0.3 1 3 10 30
HA-UbPA (ABP): - + + + + + + +
USP28-ABP
USP28
USP25-ABP
USP25
anti-
HA
anti-
GAPDH

C

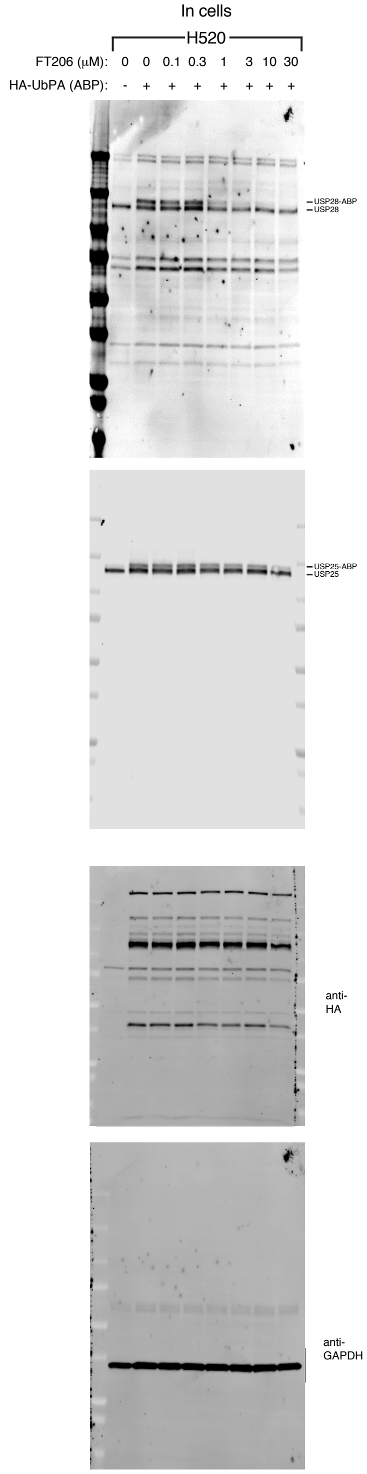
In cells
H520
FT206 (μM): 0 0 0.1 0.3 1 3 10 30
HA-UbPA (ABP): - + + + + + + +
USP28-ABP
USP28
USP25-ABP
USP25
anti-
HA
anti-
GAPDH

E

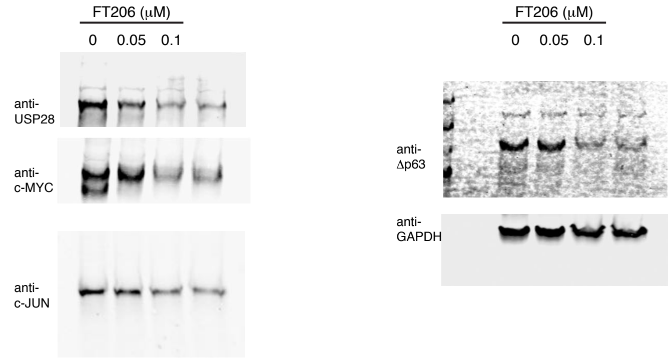
FT206 (μM)
0 0.05 0.1
anti-
USP28
anti-
c-MYC
anti-
c-JUN
FT206 (μM)
0 0.05 0.1
anti-
Δp63
anti-
GAPDH

Figure 4

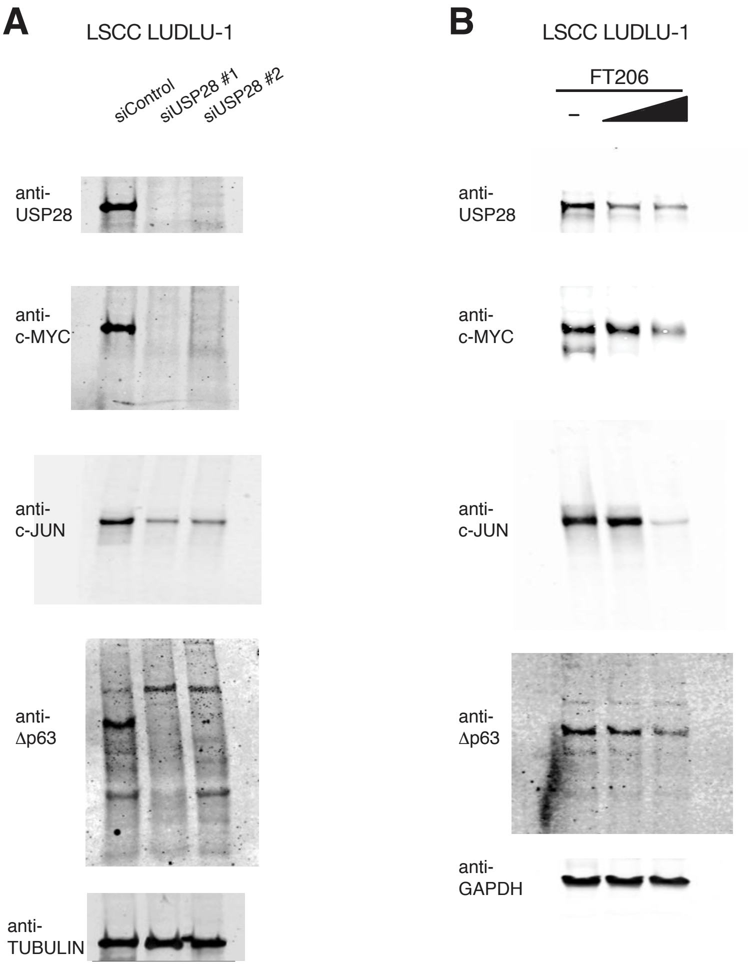
A
LSCC LUDLU-1
siControl
siUSP28 #1
siUSP28 #2
anti-
USP28
anti-
c-MYC
anti-
c-JUN
anti-
Δp63
anti-
TUBULIN
B
LSCC LUDLU-1
FT206
–
anti-
USP28
anti-
c-MYC
anti-
c-JUN
anti-
Δp63
anti-
GAPDH

Figure 6

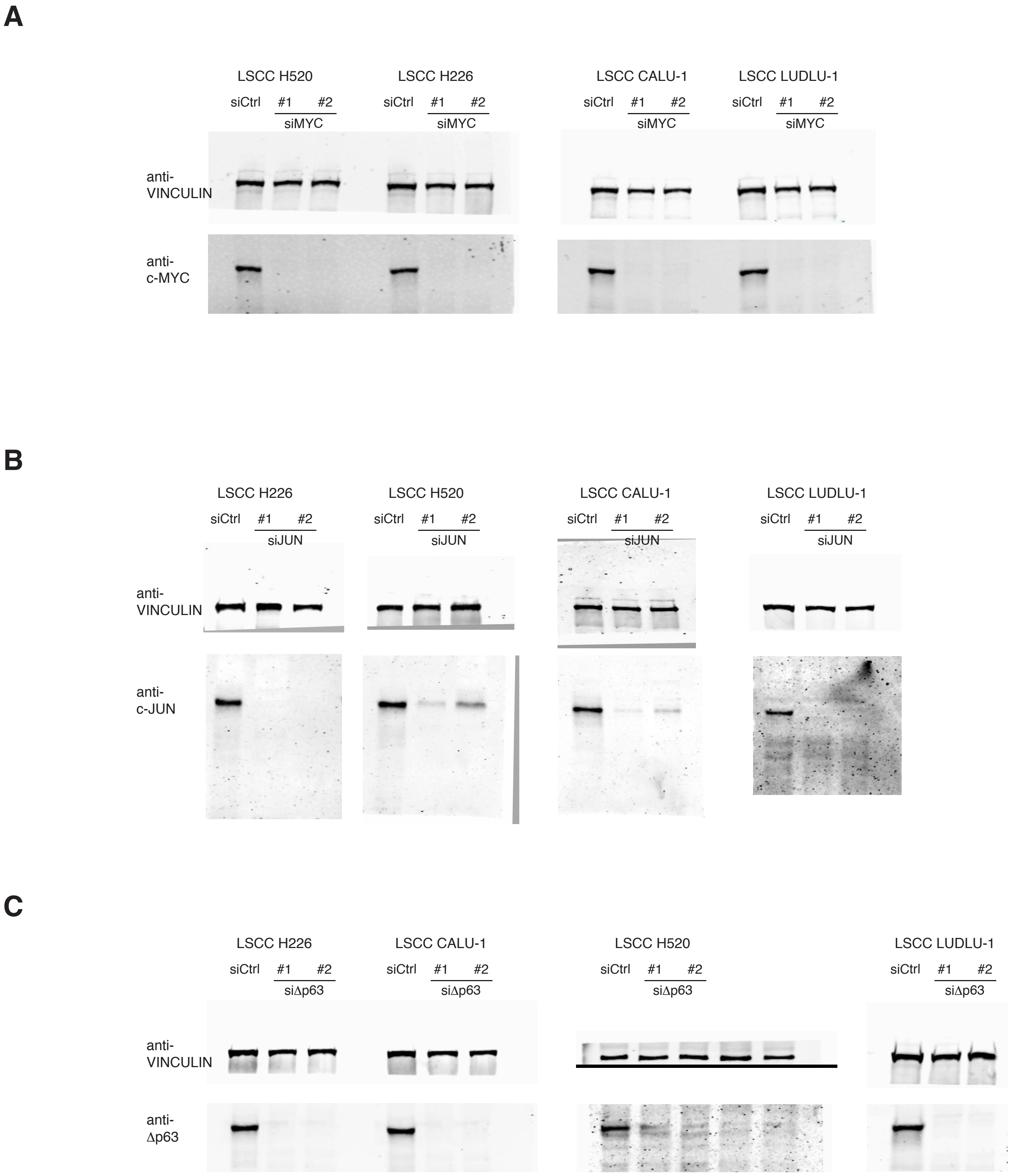

Figure 1 – figure supplement 1

A

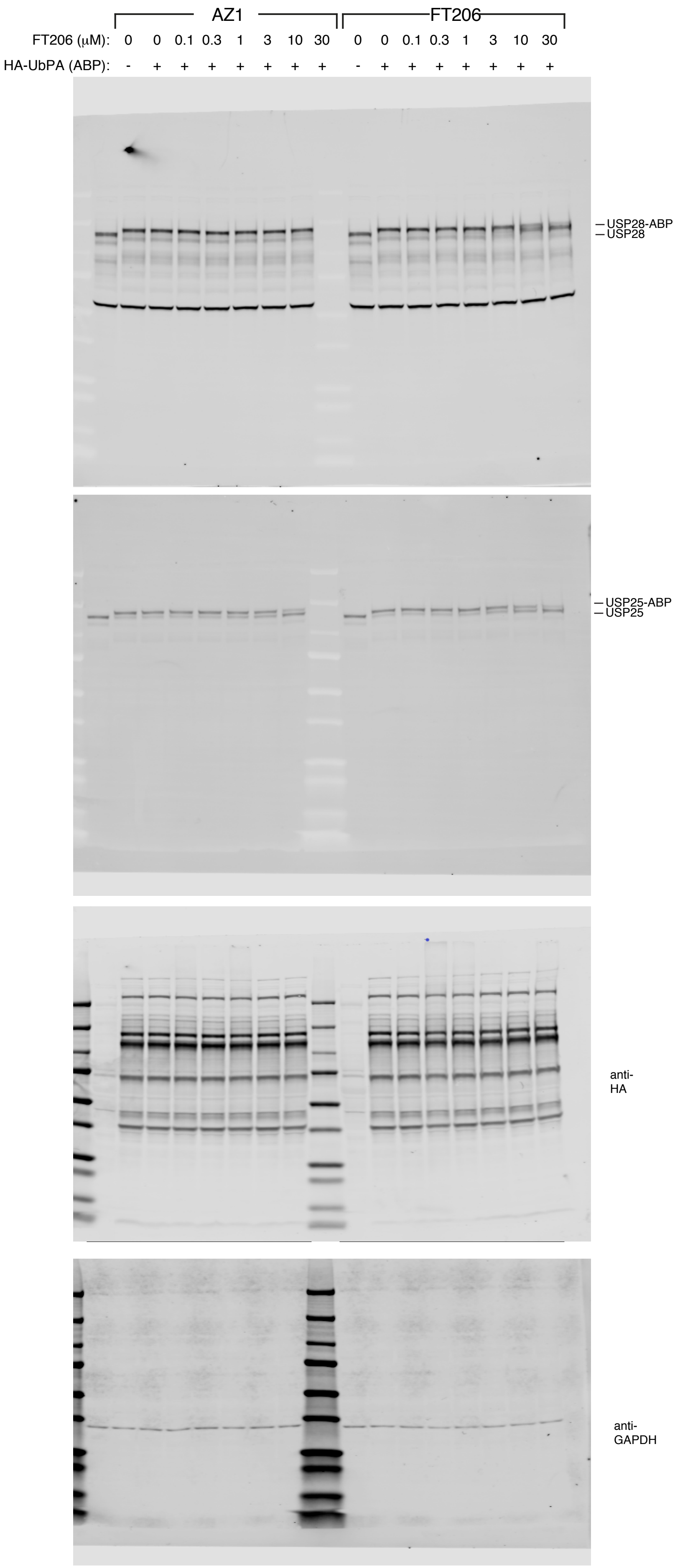

Figure 4 – figure supplement 1

B

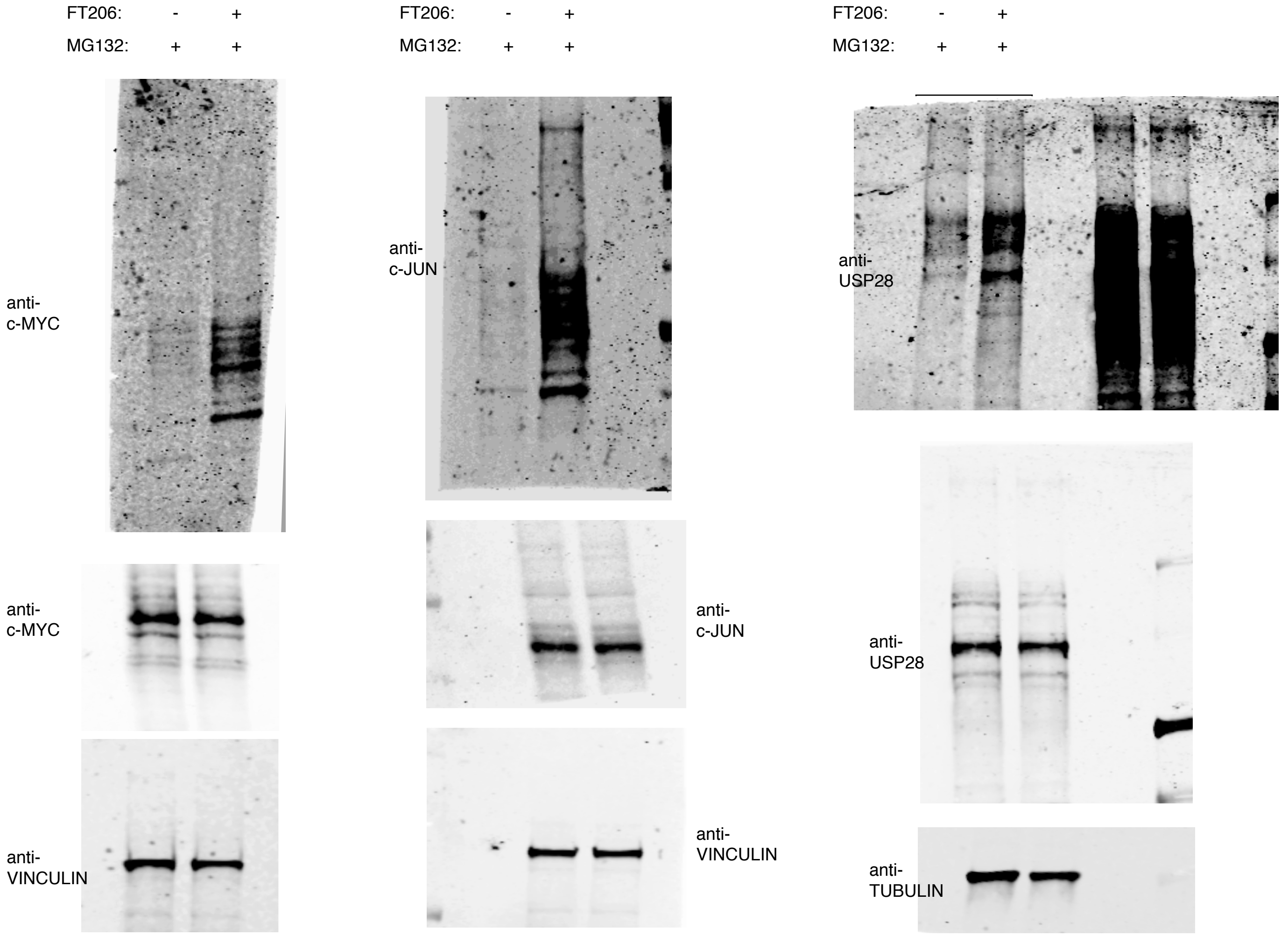

C

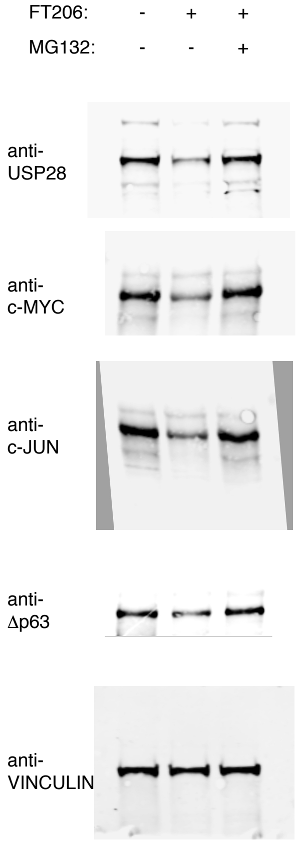

D

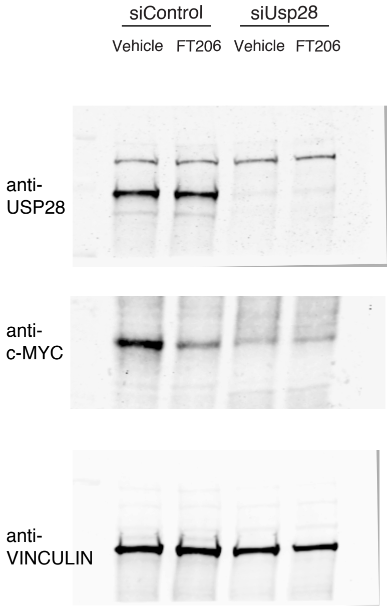

Figure 4 – figure supplement 1

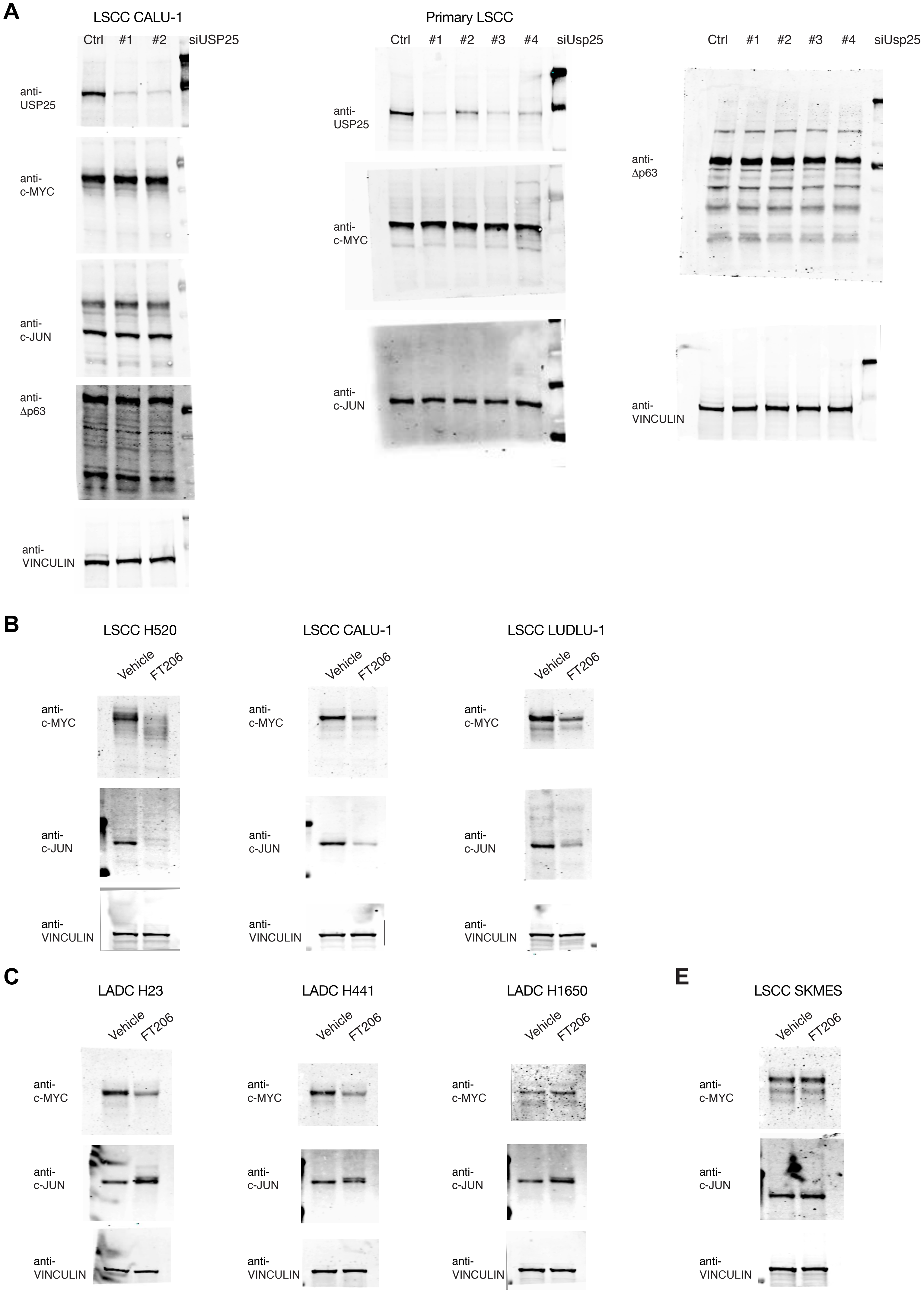

Figure 6 – figure supplement 1
