# Supplementary material for: USP28 deletion and small-molecule inhibition destabilizes c-MYC and elicits regression of squamous cell lung carcinoma
Source: eLife. 2021 Oct 12;10:e71596. doi: 10.7554/eLife.71596 (PMC8553340; doi:10.7554/eLife.71596)
Supplement: Transparent reporting form [file elife-71596-transrepform1.pdf]

Westbrook Centre, Milton Road
Cambridge CB4 1YG
UK
P 01223 855340
W elifesciences.org
T @elife

eLife's transparent reporting form

We encourage authors to provide detailed information *within their submission* to facilitate the interpretation and replication of experiments. Authors can upload supporting documentation to indicate the use of appropriate reporting guidelines for health-related research (see EQUATOR Network), life science research (see the BioSharing Information Resource), or the ARRIVE guidelines for reporting work involving animal research. Where applicable, authors should refer to any relevant reporting standards documents in this form.

If you have any questions, please consult our Journal Policies and/or contact us: editorial@elifesciences.org.

Sample-size estimation

- You should state whether an appropriate sample size was computed when the study was being designed
- You should state the statistical method of sample size computation and any required assumptions
- If no explicit power analysis was used, you should describe how you decided what sample (replicate) size (number) to use

Please outline where this information can be found within the submission (e.g., sections or figure legends), or explain why this information doesn't apply to your submission:

The sample size (n) and the statistical method are indicated in the figure legend, or in the Materials and Methods section.
No power analysis was applied. Knowing that a heterogeneity exists between mice, cohorts of 3 or more animals per condition were used, which generates enough power to pick up statistically significant differences between treatments, as determined from previous experience (J Exp Med 216, 450-465).

Replicates

- You should report how often each experiment was performed
- You should include a definition of biological versus technical replication
- The data obtained should be provided and sufficient information should be provided to indicate the number of independent biological and/or technical replicates
- If you encountered any outliers, you should describe how these were handled
- Criteria for exclusion/inclusion of data should be clearly stated
- High-throughput sequence data should be uploaded before submission, with a private link for reviewers provided (these are available from both GEO and ArrayExpress)

Please outline where this information can be found within the submission (e.g., sections or figure legends), or explain why this information doesn't apply to your submission:

A general statement is given in the material and methods section (statistics), the exact number of biological replicates is provided in the figure legends.

eLife Sciences Publications, Ltd is a limited liability non-profit non-stock corporation incorporated in the State of Delaware, USA, with company number 5030732, and is registered in the UK with company number FC030576 and branch number BR015634 at the address Westbrook Centre, Milton Road Cambridge CB4 1YG, UK | March 2019

Statistical reporting

- Statistical analysis methods should be described and justified
- Raw data should be presented in figures whenever informative to do so (typically when N per group is less than 10)
- For each experiment, you should identify the statistical tests used, exact values of N, definitions of center, methods of multiple test correction, and dispersion and precision measures (e.g., mean, median, SD, SEM, confidence intervals; and, for the major substantive results, a measure of effect size (e.g., Pearson's r, Cohen's d)
- Report exact p-values wherever possible alongside the summary statistics and 95% confidence intervals. These should be reported for all key questions and not only when the p-value is less than 0.05.

Please outline where this information can be found within the submission (e.g., sections or figure legends), or explain why this information doesn't apply to your submission:

For each experiment, all data related to statistical reporting are indicated in the figure legend or in the Materials and Methods section. We showed the exact p-values on graphs. We present raw data (dot plots) where relevant.

(For large datasets, or papers with a very large number of statistical tests, you may upload a single table file with tests, Ns, etc., with reference to sections in the manuscript.)

Group allocation

- Indicate how samples were allocated into experimental groups (in the case of clinical studies, please specify allocation to treatment method); if randomization was used, please also state if restricted randomization was applied
- Indicate if masking was used during group allocation, data collection and/or data analysis

Please outline where this information can be found within the submission (e.g., sections or figure legends), or explain why this information doesn't apply to your submission:

This information is provided in the Materials and Methods section.

Additional data files ("source data")

- We encourage you to upload relevant additional data files, such as numerical data that are represented as a graph in a figure, or as a summary table
- Where provided, these should be in the most useful format, and they can be uploaded as "Source data" files linked to a main figure or table
- Include model definition files including the full list of parameters used
- Include code used for data analysis (e.g., R, MatLab)
- Avoid stating that data files are "available upon request"

Please indicate the figures or tables for which source data files have been provided:

The figures for which source data files have been provided are the following:
Figure 1: Figure1B, Figure1D, Figure1E, Figure1G and Figure1I
Figure 2: Figure2C, Figure2D, Figure2F and Figure2G
Figure 3: Figure3C and Figure 3D
Figure 4: Figure 4D, Figure4F
Figure 5: Figure5C, Figure5D, Figure5E, Figure5F and Figure5I
Figure 6: Figure6C, Figure6D, Figure6E, Figure6F, Figure6G, Figure6H and Figure6I
Figure S1: FigureS1A, FigureS1B and FigureS1C
Figure S2: FigureS2A
Figure S4: FigureS4D
Figure S5: FigureS5A
